# Using Human-Centered Design to Adapt Supply Chains and Digital Solutions for Community Health Volunteers in Nomadic Communities of Northern Kenya

**DOI:** 10.9745/GHSP-D-20-00378

**Published:** 2021-03-15

**Authors:** Sarah R. Andersson, Sarah Hassanen, Amos M. Momanyi, Danielson K. Onyango, Daniel K. Gatwechi, Mercy N. Lutukai, Karen O. Aura, Alex M. Mungai, Yasmin K. Chandani

**Affiliations:** a Murdoch Children's Research Institute, Melbourne, Australia.; b Human-Centered Design Corner, Nairobi, Kenya.; c inSupply Health Limited Kenya, Nairobi, Kenya.; d Ministry of Health Republic of Kenya, Nairobi, Kenya.

## Abstract

**Background:**

Unreliable and nonexistent supply chain procedures and processes are one of the primary barriers to achieving functional community health units in nomadic communities in the arid/semiarid counties of Kenya.

**Methods:**

We used a human-centered design (HCD) approach to engage communities and community health volunteers (CHVs) in redesigning a proven data-centric supply chain approach that included a digital solution, called cStock, for this challenging context. We conducted the HCD process in 4 phases: (1) understanding intent, (2) research and insights, (3) ideation and prototyping, and (4) supply chain design and requirements building. Data collection used qualitative methods and involved a range of stakeholders including CHVs, supervisors, and local beneficiaries. CHVs and their supervisors also participated in cStock usability testing. Drawing on insights and personas generated from the research, stakeholders ideated and codesigned supply chain tools.

**Results:**

The research identified critical insights for informing the redesign of cStock for nomadic communities. These insights were categorized into supply chain, information systems, human resources, behaviors, service delivery infrastructure, and connectivity. Four supply chain data solutions were designed, prototyped, tested, and iterated: a stock recording paper-based form, a user-friendly cStock application, a supervisor cStock application, and an unstructured supplementary service data reporting system using feature phones.

**Conclusions:**

Using the HCD process incorporated the perspective of CHVs and their communities and provided key insights to inform the design of the supply chain and adapt cStock. The process helped make cStock to be inclusive and have the potential to have a meaningful impact on strengthening the supply chain for seminomadic and nomadic communities in northern Kenya. A strong supply chain for these CHVs will increase access to essential and reproductive health commodities and contribute to improving the overall health and well-being of these communities, especially women and children.

## Abstract (precis)

Investing the time and effort to use human-centered design (HCD) approaches is beneficial to designing supply chains and digital solutions for complex sociocultural settings. HCD enables users to be engaged in cocreating solutions that address their challenges, are appropriate for their context and capacity, and build local ownership.

## Teaser

**Key Findings:**

- Human-centered design (HCD) offers a methodology for engaging seminomadic and nomadic communities with complex social and cultural barriers in designing innovative digital health and supply chain solutions. A reliable supply chain for essential and reproductive health commodities for community health volunteers will improve access and contribute to the overall health and well-being of the community, especially women and children.
- Exploratory research using HCD methodologies uncovered opportunities for innovation by building on informal behaviors already in place to overcome barriers, such as strengthening the role of supervisors to support community health volunteers in completing their reports.

**Key Implications:**

- Program managers should consider using HCD approaches when designing health, digital, or supply chain interventions for complex sociocultural settings, especially when traditional health interventions have not been successful.

## INTRODUCTION

**FIGURE 1:**
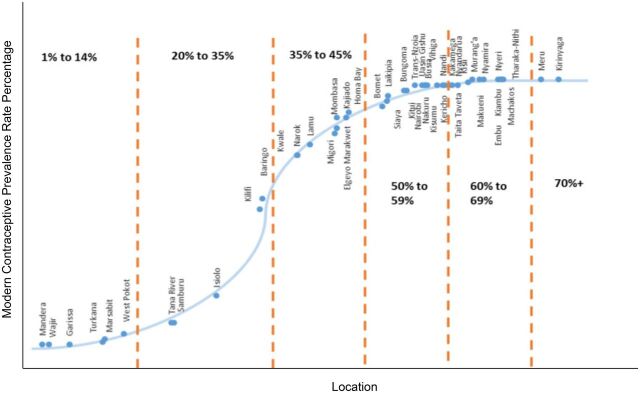
S-Curve for Modern Contraceptive Prevalence Rate in Kenya, by County^a^ ^a^ From the 2014 Kenya Demographic and Health Survey.

Community health volunteers (CHVs) in Kenya play a critical role in improving the health of populations and extending health care to communities.^1^ Kenya's progress in improving reproductive health outcomes have not been consistent across all of its 47 counties, as depicted in Figure 1.^2^ Modern contraceptive prevalence rate (mCPR) in the northern arid/semiarid lands (ASAL) inhabited by nomadic communities is still very low, ranging from 2% to 10%.^3^

Seminomadic and nomadic people face multiple barriers to health care access, including geographic isolation, lifestyle, sociocultural dynamics, and logistical, political, and economic factors.^4^ Providing health care to nomadic people is known to be extremely complex, with barriers that include both external (geographic isolation, sociocultural dynamics, and logistical and political factors) and internal factors (lifestyle, norms, practices, and perceptions). A systematic review by Ali et al. identified a combination of fixed and mobile services can be effective in addressing the reproductive health needs of nomadic populations.^5^ The introduction of volunteer CHVs within these populations can improve the connections between nomadic communities and the health system and facilitate their access to modern health systems, especially reproductive health care.^6^

In recent years, the Kenyan Ministry of Health and county governments have been working with other nongovernmental organizations (NGOs) to strengthen the design of community health units for nomadic communities in the northern ASAL counties of Kenya, as part of the overall community health strategy. This strategy focuses on providing girls and women with family planning and reproductive health services to address the inequity in reproductive health outcomes, such as the low mCPR (Figure 1). However, the success of these community health units depends on sustainable, regular access to health commodities.^7^^,^^8^ NGOs supporting the government to strengthen community health services in the ASAL communities identified that unreliable and nonexistent supply chain procedures and processes are one of the primary risks to achieving a functioning community health unit. The Supply Chain Alternatives for Last Mile Equity (SCALE) project, described in this article, was designed to address this risk by designing sustainable, scalable supply chain solutions for the unique challenges of this population in line with the county government efforts to improve the health and well-being of these communities through a community health strategy that is specific to the needs of this population.

Unreliable and nonexistent supply chain procedures and processes are one of the primary risks to achieving a functioning community health unit.

Community health programs globally are often hindered by poor availability of health commodities.^9^ Evaluations across multiple Eastern and Southern African countries shows that community health supply chains are rarely designed deliberately or purposefully for the community level context and often lack appropriate procedures for inventory management, replenishment, and data recording and reporting. Often there is little to no visibility at the national or subnational levels into stock levels at the community level, and data are not used for decision making, which leads to inadequate stock for community-based distribution, facility level stock-outs, and product wastage.^10^

Previous experience in designing supply chains for community health workers found that the “gold standard” is a supply chain design that combines a demand-based resupply with real-time reporting, such as a mobile system that makes stock data visible to all levels of the health system simultaneously to enable rapid decision making. The findings also highlight the importance of strengthening capacity to use the data for monitoring supply chain performance and promoting local problem solving based on data through the implementation of data review meetings.^10^

The SCALE design team identified cStock, a proven supply chain approach for CHVs, as a starting point for designing a system for these nomadic CHVs. Originally implemented in Malawi, cStock was later redesigned for Siaya County, Kenya (Box 1). The approach combines a simple digital solution that is flexible enough to support areas of low cellular network and offline data entry.^11^^,^^12^ The tool is designed to support supply chain best practices, such as demand-based resupply, and provides dashboards to facilitate system-wide data visibility and transparency. The approach also includes data review meetings that strengthen the use of data and local problem solving for supply chain challenges.^11^

BOX 1 **cStock in Malawi and Kenya**

In Malawi, cStock was introduced in 2010 and is a simple short message service (SMS)-based reporting and resupply system. CHVs report stock data through their personal mobile phones to a web-based dashboard, where resupply quantities are automatically calculated based on past consumption and a request is sent to supervisors.

In Siaya County, Kenya, in 2017, cStock was redesigned to include a smartphone application as well as SMS, as smartphone use increased across the region. CHVs are able to use either method for reporting data depending on their access to smartphones and data coverage. cStock was embedded within the national health management information system, and data can be accessed on dashboards at all levels of the health system.

Recognizing that the context in the northern ASAL counties of Kenya was likely to be vastly different from the context in which cStock has previously been implemented, we considered redesigning cStock with this new group of users to be critical to the project's success. The design team expected the differences to include lower literacy including digital literacy, migration interrupting access and timing of resupply, and unknown social and cultural behaviors that could impact CHVs' relationship to their supervisors and the health system that might impact the supply chain.

Human-centered design (HCD) offered an approach that could help the design team understand and address the social and cultural barriers by engaging the CHVs and these communities in the redesign. HCD sees users as an essential source of knowledge of their lives and contexts and emphasizes iterative end-user involvement during all stages of the design process. The HCD approach enabled the design team to understand how to incorporate supply chain best practices into a community health model for remote areas and adapt the design of digital technology solutions for this challenging and complex context.

The HCD approach enabled the design team to understand how to incorporate supply chain best practices into a community health model for remote areas and adapt the digital technology solutions for this complex context.

## METHODS

**FIGURE 2:**
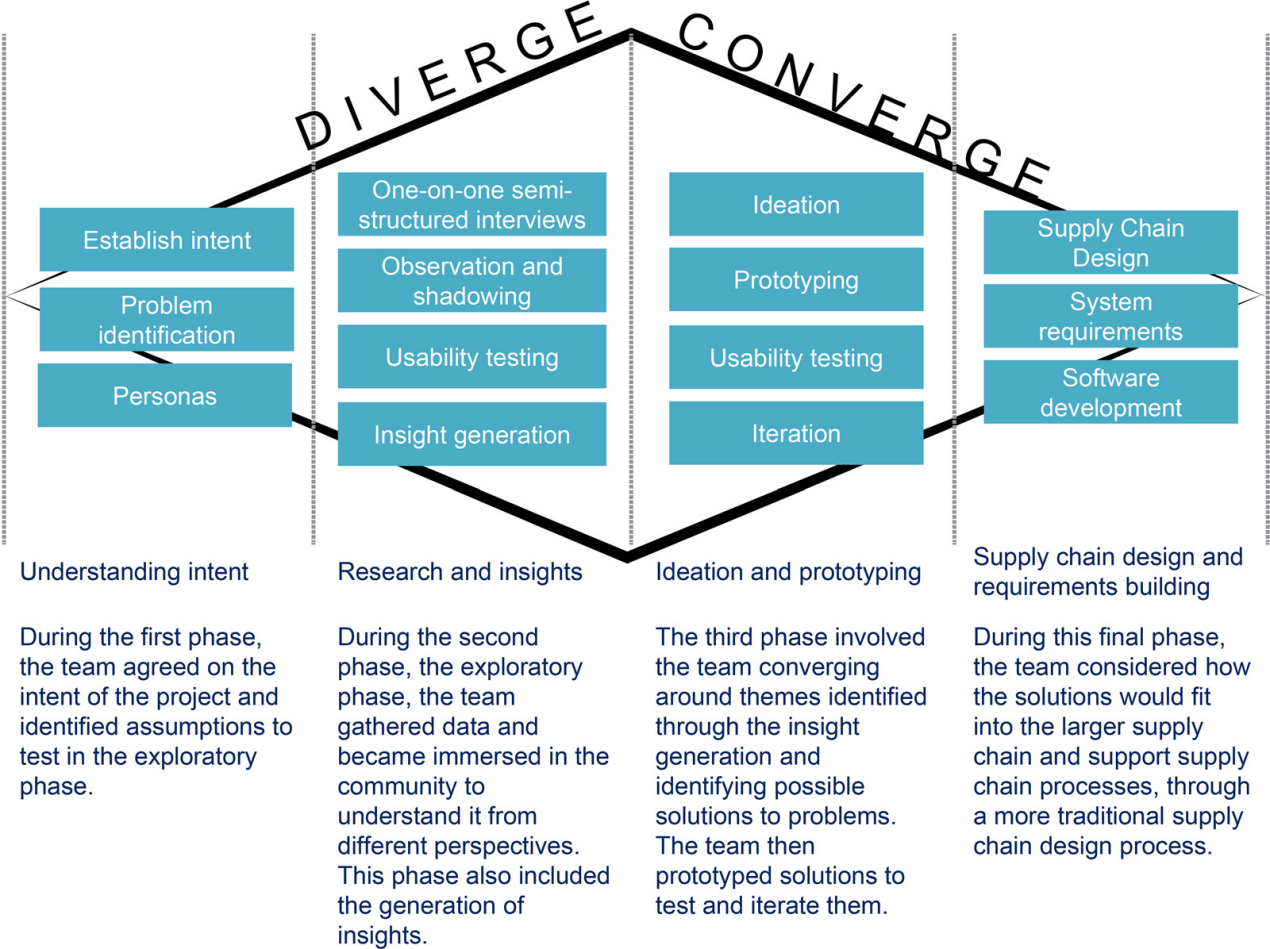
Human-Centered Design Methodology

HCD is not explicitly a research methodology but incorporates a series of mixed qualitative methods to achieve design objectives.^13^ HCD is a multistage problem solving process that requires designers to conduct research to understand the users and subsequently test solutions in real-world scenarios with actual users. The HCD process for this 3-year project took more than 7 months (February to August 2019) in 4 phases: (1) understanding intent, 1 month; (2) research and insights, 3 months; (3) ideation and prototyping, 2 months; and (4) supply chain design and requirements building, 1 month (Figure 2). Recognizing the context was challenging, we expected from the project's outset that the first year would be dedicated to designing the intervention and that it would take time to understand and design a suitable system.

### 1. Understanding Intent

The intent phase resulted in a 1-page intent statement, documenting the shared understanding among the design team of the broad questions, objectives, target groups, approach, risks, and aims. The intent identified was to design a supply chain and digital solution that addresses the CHV challenges, facilitate their commodity management responsibilities, and recognize the broader cultural and societal beliefs, norms, and rituals. This statement was used as a living document and iterated upon throughout the project.

Then, the design team developed personas based on our target community for this project: CHVs, women of reproductive age, community leaders, and husbands. The design team was primarily composed of Kenyans with representation from our target regions. Through the personas, the design team captured what they thought they knew about the motivations, challenges, and beliefs of the community based on their personal experience. The personas were used to create lines of inquiry for the research, and research guides and tools were designed for each persona group. The design team continued to iterate on the personas throughout the research phase and refine their personas during their insight generation sessions to ensure they reflected the types of people they talked to (Box 2).

BOX 2 **Description of Personas**

Personas are fictitious characters who bring to life the needs, goals, values, drivers, and behaviors of larger groups of people; they can only represent groups and behaviors, and as such they are composite images of real user groups.

Personas build deep empathy and understanding of the potential user groups and help define the design considerations or criteria that need to be explored. A persona is a living tool that serves as a hypothesis of assumptions; research tools can be developed to check those assumptions.

### 2. Research and Insights

Data were collected in 2 of the 4 project counties, Wajir and Samburu. The research centered on 4 objectives to understand: (1) infrastructure, access, capabilities, and the current state of the health system; (2) decision making, knowledge, and sources of influence; (3) gender dynamics and cultural norms; and (4) technology awareness, capabilities, preferences, and usage.

A combination of methods was used, including one-on-one semistructured interviews; observation and shadowing of supply chain actors, end-users, and county beneficiaries; and usability testing using the original cStock application from Siaya County. For both interviews and observations, a mixture of research guides and user mapping activities was used to help generate deeper insights.

We conducted interviews with 1 national MOH representative, 34 people in Samburu Central and East, and 27 people in Wajir Central and East (Table 1). The interviews were conducted in a location of the participant's choosing, such as health facilities, CHVs' houses, and walking with CHVs during community visits. Interviews began with trust-building techniques such as asking personal questions. Open-ended questions were used to discuss job requirements and challenges, supply chain challenges, health behaviors and preferences, and social networks and preferences in communication. Interviews also incorporated storytelling to bring out the nuances of the participants' lives, work, and motivators. “Day in the life” mapping tools were used; these included templates to map out daily routines and network mapping templates to map important nodes within their social networks.

**TABLE 1.** Participants Interviewed for Research on Designing Supply Chain Solution for Community Health, 2 Counties in Kenya

	Samburu Central and East County (N=34), No.	Wajir Central and WestCounty (N=27), No.
County and subcounty health management team members	7	12
Health care workers including CHVs, CHV supervisors, health facility in-charges, nurses, and family planning coordinators	8	10
Project partners	2	2
Community members including WRA, matriarchs, husbands of WRAs, and community and religious leaders	17	5

Abbreviations: CHV, community health volunteer; WRA, women of reproductive age.

Observation was used to validate the findings from interviews by noting any differences between words and actions. This provides insight into relevant behaviors that the individual could not articulate. The design team conducted immersive observations of participants within their natural environments in an unobtrusive manner for periods ranging from several hours to half or three-quarters of a day at a time. Team members followed CHVs on their community visits, including into remote mountainous regions in Samburu and seminomadic settlements in Wajir, staying in the background and taking the time to gain empathy, garner insight, and put themselves into users' shoes to incorporate their observations into the design phase. It is understood that participants likely alter behavior while being observed, however, this method in conjunction with usability testing and structured interviews can cross check data across methodologies and their limitations.

Our team also observed the usability of the original cStock app with CHVs in migratory communities. Usability testing guides with expected scenarios were developed to guide the testing. The design team loaded the original cStock application onto their personal phone and handed the phone to the CHV to observe how they navigated the application. A similar process was observed using short message service (SMS). Table 2 describes the original solutions and Table 3 outlines the scenarios used for the different rounds of usability testing.

**TABLE 2.** Four Key Supply Chain Solutions Designed for Community Health, 2 Counties in Kenya

Supply Chain Solution	Old Solution	Problems Addressed
1. **Paper-based stock record:** redesigned to be highly visual, with circles for tallying, illustrations of the product and disease/condition	Paper-based stock record used in Siaya County, Kenya, that was in English with no illustrations	CHVs' low literacy levels CHVs verbally reporting to supervisors CHVs incorrectly recording data Lack of standardized tool used by all communities to report on logistics data
2. **cStock smartphone application**: Redesign of the application that includes simplified navigation, context appropriate language, audio prompts, and visual cues	Original cStock smartphone application designed for Siaya County, Kenya	CHVs' illiteracy and language barriers CHVs' lack of understanding of supply chain terms and concepts CHVs' login challenges/user friendliness
3. **CHV supervisor smartphone application**: New cStock application for the CHV supervisors designed to be used to enter data for low literacy CHVs and to validate data plus allow for alternative supply arrangements during migration	Informal processes already in place	CHVs' with low tech capacity and low literacy levels had difficulty with data entry and data quality monitoring Supervisors previously reporting on CHVs' behalf but in a nonsystemized way
4. **USSD reporting system**: Hybrid USSD and SMS reporting structure to be used by CHVs to report on supply chain data via a feature phone, eventually reiterated to be only USSD reporting	SMS only system used in Siaya County, Kenya	Some CHVs lacking smart phones CHVs' challenges entering symbols for structured SMS option CHVs' difficulty with case-sensitive SMS structure USSD only option has a time-out feature

Abbreviations: CHV, community health volunteer; SMS, short message service; USSD, unstructured supplementary service data.

**TABLE 3.** Usability Testing Scenarios for Original Supply Chain Tools and 4 New or Redesigned Supply Chain Solutions for Community Health, 2 Counties in Kenya

Supply Chain Solutions	Usability Testing Scenarios	
	Original Tools	New or Redesigned Solutions
Paper-based stock record	Printed original form used in Siaya County, provided CHVs with mock data and asked to fill out the form with minimal instructions	Printed Excel prototypes of redesigned form, provided CHVs with mock data and asked them to fill out the form with minimal instructions
cStock smartphone application	Uploaded original cStock application onto researcher's phone. Provided CHVs with mock data and asked them to complete form with minimal instructions	Developed and uploaded clickable prototypes onto researcher's phone. Provided CHVs with mock data and asked them to complete form with minimal instructions
CHV supervisor smartphone application	Not applicable	Developed and uploaded clickable prototypes onto researcher's phone. Provided CHV supervisors with mock data and asked them to complete form with minimal instructions
USSD reporting system	Provided CHVs with mock data and job aids for SMS and asked them to follow instructions to send SMS	Provided CHVs with paper prototypes for USSD and then moved to phone to enter an example SMS

Abbreviations: CHV, community health volunteer; SMS, short message service; USSD, unstructured supplementary service data.

Data from interviews, observations, and usability testing were analyzed using a HCD technique called “insight generation” — a set of data that clusters around an observation and gives a new (or validated) perspective. For example, an insight on the CHV supervisor's role in reporting captured that CHVs verbally reported to their supervisors, who would then complete the report on the CHV's behalf; in other situations, the CHVs entered the data, but the supervisor validated the data. Through a process of rapid synthesis in the field and extended office-based synthesis, the design team “made sense” of the data by connecting and consolidating the raw data into meaningful insights. Each individual captured their observations from the field research on sticky notes, without any analysis or interpretation. The design team then found themes by grouping similar insights together. Once observations were captured into themes, the design team identified similarities and differences across the counties and developed targeted insights that guided the designers to ask questions about the design and to devise innovative strategies.

Data from interviews, observations, and usability testing were analyzed to generate insights—a set of data that gives a new perspective.

### 3. Ideation and Prototyping

A 2-day ideation and prototyping workshop was held to validate, iterate, and create solutions based on the findings. The workshop brought together 35 representatives from a variety of stakeholder groups to redesign cStock: government officials, national medical stores, county pharmacists, digital solution providers, industry experts, implementing partners, and project staff. The facilitators shared insights and the refined user personas to bring the users' voices into the process and closely reflect their needs in the design of the prototypes.

Based on the findings, the workshop participants identified 3 of the existing tools used in Siaya County required redesigning and 1 new tool needed to be designed to support supply chain data collection and strengthen supply chain monitoring and decision making. The participants used a prototype development worksheet to draw their designs. The participants were also given storyboard templates, wireframes for both unstructured supplementary service data (USSD) and smartphones, and pictures of already existing tools such as paper-based forms and the existing cStock application. Table 3 outlines the 4 solutions.

After the workshop, the design team developed higher-fidelity prototypes using different software and tools. One prototype of a paper-based stock record was developed in Excel and printed so users could be observed completing the form. For the 2 smartphone apps, clickable prototypes were developed using JustinMind software, which enables developers to create a prototype that simulates how the app would function on a phone, which in turn, enables the design team to observe the user experience.^14^ The prototype of the hybrid SMS/USSD system using feature phones (earlier-generation phones with button keypad) was illustrated on paper, and pre-populated messages were loaded on a real phone. The CHVs were asked to “press” the buttons on the paper phone, and this would lead them to different screens. To enter their data, the CHVs were instructed to enter messages in a feature phone provided by the design team. Depending on the user's choice, the design team would send an SMS of what a real message response might look like.

Usability testing was then conducted using the high-fidelity prototypes of the 4 new and redesigned tools. The testing was conducted in Samburu East in 4 rural community units that tend to migrate more often and in areas of low internet connectivity and included 27 CHV supervisors and CHVs. Table 2 shows the scenarios used in usability testing.

### 4. Supply Chain System Design and Requirements Building

The HCD research focused on paper-based and digital tools for improving supply chain data visibility, recognizing that good supply chain data for decision making are essential for a strong supply chain. In addition to using the HCD approach, it was essential to design the supply chain procedures and processes to align the flow of community-level data and health commodities. A supply chain design methodology typically includes 4 key activities: scoping, analysis, design, and implementation.^15^ HCD provided the required information for the scoping and analysis phases of supply chain design. As the supply process from the national warehouse to health facilities is well-established in Kenya, the focus was primarily on the last step in the supply chain—from health facility to CHV. The design team held a separate 2-day workshop with stakeholders including national-level representatives, county pharmacists, community health coordinators, and partner organizations. Using the personas for CHVs and facility-level staff generated earlier through the HCD process, the participants mapped how information and health products would flow from the central warehouse to the CHVs, taking into consideration any unique requirements for migration. The supply chain design provided the inventory rules needed to develop the requirements for rebuilding the cStock application.

## RESULTS

### 1. Understanding Intent

The design team agreed the intent of the research was to understand how to design a supply chain and digital solution that addressed the challenges of nomadic CHVs; facilitated their commodity management responsibilities; and recognized the broader cultural and societal beliefs, norms, and rituals. Personas were developed by the design team that included motivations, challenges, and beliefs. One example of a common assumption was that children and livestock were primary motivators for decision making in this community, and that women had a limited role in decision making especially regarding health and family planning.

### 2. Research and Insights

The research team identified insight summaries or themes that were derived from more detailed insights (Table 4). The insights are categorized into behavioral, supply chain, information system, human resources, service delivery infrastructure, and connectivity. Insights include specific insights for CHVs but also general insights for community members recognizing that CHVs are also members of the communities, and these constraints impact their ability to perform their role as a CHV.

**TABLE 4.** Human-Centered Design Insights Generated From Research on Community Health Volunteer Use of Supply Chain Tools, 2 Counties in Kenya

	Relevance to Design	Illustrative Quotes
**Behavioral Insights**		
Nomadic migration has dramatically declined due to the need for education, health, and demarcation of land.	Migration was less of an issue than originally assumed and some simple design considerations were required for resupplying CHVs during migration.	“People move in search of pastures and water. They move within the sub-county and rarely outside.” —County Health Management Team member, Wajir “Communities here no longer migrate, land is being partitioned and allocated to the community.” —CHV, Samburu
In some communities, only men move with their animals, leaving their women and children behind.	As women and children were left behind, CHVs will also remain.	“I usually migrate and leave my wives in this home. When I migrate I take 1 wife …, I usually take the wife that doesn't have any young children. I haven't migrated for 5 years.” —Husband of women of reproductive age, Samburu
Members from the community do visit nearby centers to trade and get basic supplies.	In many situations, CHVs within these nomadic communities will have access to health facilities to collect supplies even if migrating.	
Mobile phones are the main mode of communication between the people that move and the ones that are left behind.	The use of mobile phones is increasingly common and important for these communities.	
**Supply Chain Insights**		
Health facilities, which will be or are responsible for resupplying CHVs, have stock-outs and overstocks.	Stock imbalances at facilities will result in stock imbalances with CHVs.	“Sometimes they make a blind order when they are in town.” —Subcounty Pharmacist, Samburu
Health facility staff do not routinely use data (demand data) to calculate supply requirements.	Health facilities need to have access and include CHV data in calculating their requests.	
Insufficient county level budget allocations result in an undersupply of commodities to facilities.	A current lack of data visibility does not support transparency and accountability.	“There is too little allocation of funds to commodities, and this is demoralizing to health care workers” —Subcounty health management team member, Samburu
Family planning products are supplied to the counties from the national medical stores even though demand is low, resulting in overstocking and expiries.	When counties do not request commodities, it can result in national level programs pushing out commodities.	“The uptake of FP is not high. We do not have supply chain issues for FP. We are generally overstocked rather than understocked” —Subcounty health management team member, Wajir
**Information System Insights**		
Lack of standardized supply chain reporting forms has resulted in ad hoc processes.	Standardized recording and reporting of supply chain data is critical for making data available to higher levels.	“I record commodities like condoms in a notebook, but other reports I do are MOH 513, 514, and MOH 100.” —CHV, Samburu
Service-related forms were in place but poorly used due to their complexity and unavailability.	Reporting forms must be suitable for low literacy CHVs to ensure complete and timely reporting.	“Some of the CHVs know how to read and write. The ones that don't know how come to me, and I do it for them (reporting) like their end of month report.” —CHV supervisor, Wajir
Some county and subcounty level but very few facility-level staff have skills to visualize and analyze data.	Facility staff need to be trained to use the data reported by CHVs to inform resupply and ensure CHVs get the commodities they need.	
**Human Resource Insights**		
CHVs have inadequate supervision due to lack of logistical support for supervisors to travel.	The use of mobile phones is one way to overcome some of the challenges associated with providing one-on-one supervision.	“I do supervision based on priority or challenges like availability of motorcycle or fuel. It's only my own initiative. I just support myself.” —CHV supervisor, Samburu
Lack of support throughout the system demotivates CHVs, leading to high attrition.	If CHVs are demotivated, it is essential to design a solution that makes their job easy and more rewarding. Also, take into consideration that if attrition is high, supply chain tools must be simple so new staff can be easily trained.	“We started with 20 CHVs but only 2 of them are active.” —CHV, Wajir
**Service Delivery Infrastructure Insights**		
Mobile clinics are designed to be located at strategic points such as the watering points; however, they are not operational or fully utilized due to inadequate funding and support.	Mobile clinics are being strengthened and could possibly resupply CHVs in the future.	“The nomadic clinics go to static settlements where there are no dispensaries. If they were designed differently, they would reach the nomads.” —Subcounty health management team member, Wajir
Meetings are held regularly and with high turnout throughout the system in one county but not the other.	Monthly meetings should be strengthened as part of the supply chain strategy to improve communication, data use, and local problem solving.	“During the monthly CHV meeting we plan and schedule all activities of the month since it is a challenge to send SMS in between.” —CHV supervisor, Samburu
**Connectivity Insights**		
Lack of connectivity and lack of power to charge phones hinders CHVs' communication among themselves and with their supervisor.	The design must consider that CHVs will likely not have their phones charged and have network at all times. Therefore, use of phones should be limited as much as possible to one report per month.	“Most [CHVs] have feature phones because of the battery life. Some use solar powered charges. They send 1 rep to town to charge all the phones.” —Partner, Wajir
Few CHVs had smartphones issued by partners, but struggled to use them for anything other than voice communication with family and friends.	Smartphone availability is increasing but capacity among CHVs to use them is still low. cStock should be designed for the future of smartphones.	“There is no connectivity here in the hospital, not even Safaricom or Telkom. If you want Telkom you go for about 500 meters to access it, for Safaricom you have to go to that hill (about 1 KM).” — Health facility in-charge, Samburu “Although CHVs have smartphones we usually (and they prefer too) sending SMSs”—Health facility staff, Samburu

Abbreviations: CHV, community health volunteer; MOH, ministry of health; SMS, short message service.

#### Behavioral Insights

Behavioral insights played an important role in understanding migration behaviors across the 2 counties, elucidating similarities and differences. Migration behaviors are a key consideration for the design of the supply chain and the digital tools as it is important to design for the different locations where a CHV might be based. Communities in both contexts migrate in search of water and pastures. In Samburu, however, men move with their animals, leaving their women and children behind, meaning that CHVs will also likely stay behind. In Wajir, nomadic communities live in isolation from the general population; and once a decision to migrate is made, the whole community moves and establishes a settlement in the new location. Members from the community do visit nearby centers to trade and get basic supplies, offering opportunities to also collect health products. Nomadic migration has dramatically declined as members from the communities have children in school and take on casual jobs to earn a living. Mobile phones are the main mode of communication between the people who move and those left behind. Insight into health-seeking behavior explained that among nomadic communities, visiting health facilities is a collective (and rarely women-led) decision, requiring community endorsement before the visit to the facility. Insights into health-seeking behavior are noted; although they are less important to the design of digital solutions, these insights highlighted the importance of CHVs especially regarding accessing family planning for the women in this community.

#### Supply Chain Insights

These insights described key drivers and causes of stock imbalances—under- and overstocking—at health facilities that will be or are responsible for resupplying CHVs. One reason for stock imbalances at the health facility is that staff were not always using past logistics data (consumption and stock on hand) to calculate supply requirements, and as a result, the county pharmacists reported that they do not always trust the accuracy of the report.

A reason for low stocks was insufficient county-level budget allocations that resulted in an undersupply of commodities to facilities. Some overstocking of contraceptives was explained as being due to low demand, which sometimes resulted in expiries. These products are considered public health priority products and provided free (counties do not have to purchase them) and have in the past been distributed to the counties from the central stores regardless of demand. As partners work to address the factors behind low demand, the supply chain needs to be ready with good data visibility to respond to changing demand.

#### Information System Insights

Information system insights described poor use of national reporting tools for both service and supply chain data. Low literacy combined with the complexity of tools and insufficient quantities of forms resulted in low levels of reporting. Oral reporting is common, and CHVs rely on their children to help complete reports. Currently, no standardized CHV supply chain reporting tool exists that all communities use to report logistics data. The capacity to analyze and visualize data was inconsistent at supervisory and management levels of the system. Counties do not have the budget and capacity to maintain separate information systems and use the national health information system.

#### Human Resource Insights

Human resource insights reflected both common challenges across the public health system and those exacerbated by or unique to ASAL counties. Norms and standards in recruitment policies and processes were inconsistently applied. Both ASAL counties were characterized by severe shortages and inadequate distribution of health care workers at facility and community levels. Human resource shortages are exacerbated during the rainy seasons when the communities migrate closer to the towns, resulting in long queues at health facilities and burnout among health care workers. High attrition among CHVs, who receive inadequate support and stipends, affects service provision at the community level. In both counties, CHV training often relies on externally funded activities, and recognition and award systems are in place but do not always function, even for health facility staff.

#### System-Related Insights

System-related insights described the reality of current structures and where opportunities and gaps existed. Mobile clinics have been set up in both counties to support nomadic and seminomadic communities at strategic locations, such as community watering points. At the time of research, most clinics were either not operational or not fully used due to inadequate funding and support. However, efforts to strengthen these clinics is underway and could supply CHVs with supplies in the future. In contexts where communication is constrained by distance, connectivity, and other inhibiting factors, meetings tend to happen with or without external partner support. However, community-level meetings may occur irregularly due to connectivity challenges and the distances CHVs must travel.

#### Connectivity Insights

Connectivity insights showed that 63% of the CHVs engaged during the first round of usability testing in the research phase had or regularly used a feature phone, 27% used a smartphone, and 9% did not use any type of phone. It should be noted this project did not include funds for procurement of devices so only existing devices could be used. Network connectivity was poor, and electricity to charge phones was sporadic. Many CHVs would travel to the nearest urban centers and facilities to charge their phones. Some facilities had no access to telecom services at all. Use of WhatsApp was high among facility staff, but they reached CHVs via phone calls. A few CHVs had smartphones issued by partners but struggled to use them for anything other than voice communication with family and friends.

#### Usability Testing Insights

Learnings evolved through the iterative process for usability testing (Table 2). During the research phase, the design team conducted usability testing using the original cStock application. Findings showed that the original app was not intuitive. Users struggled to navigate the multiple complicated steps and required guidance. CHVs had limited understanding of English and Kiswahili, and so they did not understand the language used in the app. Users had even more challenges with the SMS version of cStock and struggled to write structured messages that required letters, numbers, and symbols for sending reports.

We observed that usability varied by literacy levels among the participants. Literate CHVs were able to use the application, whereas those with low literacy struggled. Literacy levels also determined CHVs' ability to complete paper-based tools and understand supply chain terminology. CHV supervisors and health facility staff demonstrated higher technology capabilities but had limited knowledge of the supply chain and ability to interpret simple data visuals, such as graphs and tables.

### 3. Ideation and Prototyping

The ideation and prototyping workshop resulted in 4 principles for design.

1. For the system to be sustainable, it had to be applicable for all CHVs across Kenya; these counties alone do not have the capacity or budget to maintain the software. CHVs would need to use personal phones as procuring devices was not possible; county budgets are limited and maintaining broken or lost devices over the long term was not feasible.
2. Poor connectivity and limited power sources would not allow real-time data entry. Thus, the solution could not be entirely digital, and paper-based tools would be necessary. However, the paper-based forms could be designed to be more intuitive and appropriate for the variations in skill levels.
3. Illiteracy was high among these CHVs in these counties, and supervisors needed to be able to play a more active role in supporting CHVs.
4. The digital solution needed to be as intuitive as possible and should not require major changes to behaviors and attitudes.

**FIGURE 3:**
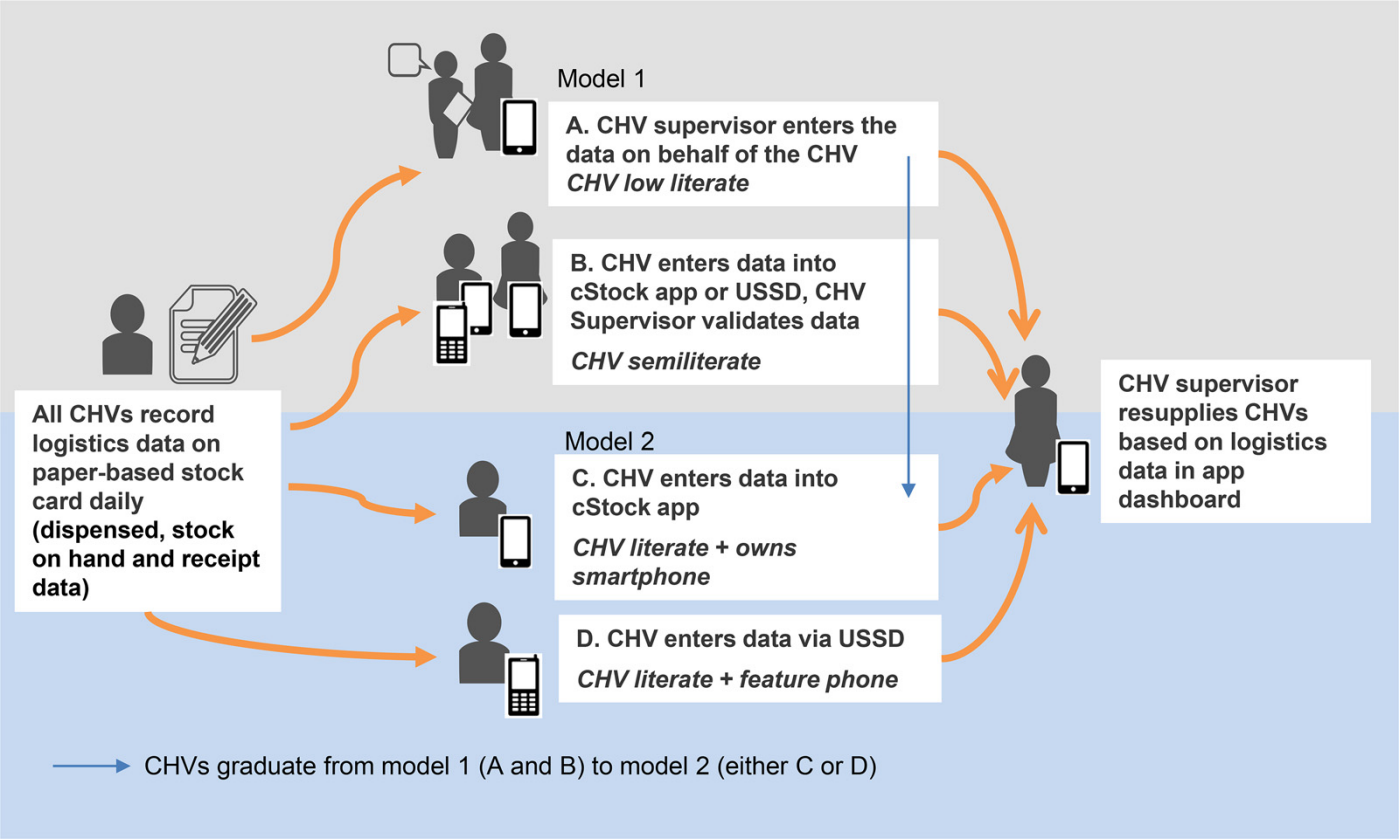
Two Models for Logistics Data Flow from Community Health Volunteers to Supervisors Abbreviations: CHV, community health volunteer; USSD, unstructured supplementary service data.

The workshop also led to the development of 2 models for data flow and 4 supply chain tools to improve data availability and use. In Model 1, supervisors enter or validate supply chain data on CHVs' behalf, using a supervisor's cStock application. In Model 2, CHVs with sufficient literacy enter the data directly into a smartphone application or USSD (Figure 3). The participants agreed that the goal should be to graduate all CHVs from Model 1 to Model 2 over time. Model 1 would be a temporary solution. After the initial training, the CHV supervisor would use monthly meetings to conduct on-the-job training to all CHVs with an eventual goal that all CHVs will enter their data either via USSD or the application. The 4 supply chain solutions as previously mentioned were a paper-based stock recording form; a redesigned, user-friendly cStock app; a supervisor version of the cStock app; and a SMS/USSD hybrid reporting prototype (Table 3).

When high-fidelity prototypes of the supply chain solutions were developed, a second round of usability testing revealed that the redesigned smartphone application was easier to use than the previous cStock. Users found it intuitive and were able to learn how to use it relatively quickly. CHVs had the most difficulty using the SMS/USSD prototype, even when they were more familiar with a feature phone. Across literacy levels, most users found it confusing to enter data in the structured manner required to report by SMS/USSD and switch between SMS and USSD. Responding to this finding, the design team decided to remove the SMS portion of the fourth (reporting) prototype and use only USSD. The usability testing also revealed that literacy and numeracy levels were not comparable. Most CHVs could count well and found ways to communicate counted numbers even if they had low literacy.

### 4. Supply Chain Design and Requirements Building

**FIGURE 4:**
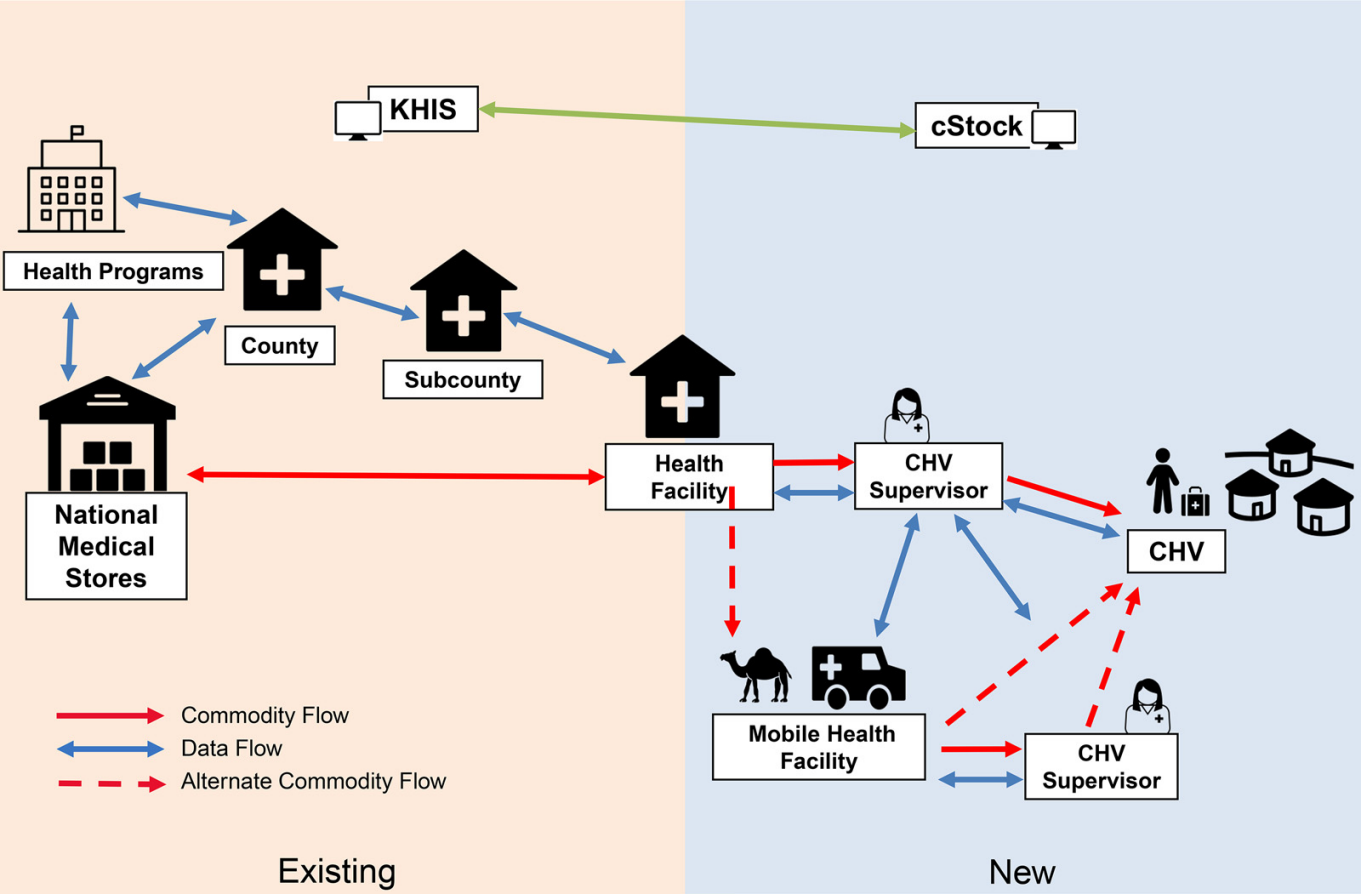
Supply Chain Design Mapping Process Showing Flow of Data and Commodities From National to Community Level, Kenya Abbreviations: CHV, community health volunteer; KHIS, Kenya Health Information System.

The supply chain design workshop yielded a standard system design mapping process from national level to community level, plus alternate processes for when CHVs were migrating. Figure 4 outlines the flow of commodities and data as mapped during the supply chain design workshop. As the supply chain processes to the health facility level are well-established in Kenya, the workshop focused primarily on the last step in the supply chain. The commodity list for the CHVs in these counties was still being discussed at the time of writing but included some basic supplies for treating worms, diarrhea, and malaria, as well as contraceptives. Two options were identified for a migrating CHV: they could receive double the quantity of commodities for the migration period or they could collect supplies from an alternative health facility during migration. CHVs could choose depending on what was more convenient: carrying more supplies or accessing another facility. Approval for the alternative arrangement rested with the CHV supervisor. The requirements for the application were developed to allow the supervisor to adjust the settings in the application to account for the change.

## DISCUSSION

HCD approaches facilitated the design of an innovative supply chain and digital solution to address challenges faced by CHVs in hard-to-reach areas and nomadic communities with complex sociocultural factors and to support the county government in improving access to health care and the overall health and well-being of these communities.^13^ Common methods used for supply chain design might not have achieved the insights and empathy gained from the HCD process and the structured, cocreative process used for translating findings into feasible, acceptable solutions. Traditionally, supply chain assessments primarily look to health care providers as their source of information.^16^ Insights generated from interacting with a variety of stakeholders, not just health care providers, highlighted contextual barriers that affect nomadic CHVs and the recurring challenges that influence the supply chain system generally. HCD enabled the design team to observe the behaviors, daily challenges, and motivations of nomadic peoples and the relationships between CHVs and their community, supervisor, and health facility.

Insights generated from interacting with a variety of stakeholders, not just health care providers, highlighted contextual barriers that affect nomadic CHVs and the recurring challenges that influence the supply chain system generally.

HCD provided insight into the needs of the people who would benefit from the innovation and helped create novel approaches to meet these needs and deliver solutions that would work in this socioeconomic context. The research techniques used provided deep insights into this complex community. For example, interviews were conducted in a place chosen by the interviewee. This ensured that each interviewee was in familiar surroundings and felt comfortable, which facilitated more genuine, meaningful interactions and responses. Interview guides ensured coverage of important lines of inquiry, while also enabling flexibility to dive into and follow the users' stories. “Day in the life” mapping tools allowed participants to visually impart information that would have been hard for interviewees to articulate or for the design team to document as observations.

The HCD technique of cocreation supported stakeholders in a constructive, creative, and inclusive process that encouraged contributions and ideas from different perspectives. Insights from the exploratory research were critical to ensure that stakeholders truly understood the end users. These insights also informed the overarching principles for the design. The 4 design principles (addressing sustainability, connectivity challenges, illiteracy, and ensuring intuitive usability) led the design team to identify the 2 distinct models for data flow to accommodate differing levels of skills, motivations, and contextual elements within the health system in northern Kenya. Model 1, which includes the supervisor's app, built on the behavior observed during the fieldwork, where supervisors would complete or validate data on paper-based reporting for CHVs with low literacy. Model 2 was designed for CHVs who have the capacity to enter the data directly into a digital solution. The design team agreed that ultimately all CHVs would graduate to Model 2 once they had capacity for self-reporting as outlined in Figure 3.

The iterative usability testing for the 4 prototype tools facilitated continuous learning and adjustment of the design. Table 5 outlines the outcomes of the usability testing and how it changed the overall design. Usability testing is a widely used user-centered technique that evaluates a product's design and function and the degree to which it meets users' expectations and needs. The use of high-fidelity, clickable prototypes enabled early, frequent usability testing before time and money were spent on software development. For example, usability testing revealed that CHVs were able to learn how to use a simpler digital solution that used more visual cues and required less steps. The testing also demonstrated that CHVs with low literacy were numerate, which informed the design team that the solutions could include tallying of data. For the paper-based forms, the design team made adjustments during testing to ensure that CHVs understood the illustrations and could navigate the tools. Thus, this iterative, user-based process resulted in truly innovative tools that were more user-friendly, more appropriate, and more likely to be used by CHVs.

**TABLE 5.** Results of Usability Testing of 4 Prototype Supply Chain Solutions for Community Health, 2 Counties in Kenya

Supply Chain Solution	Positive Results	Negative Results	Changes Made in Final Iteration
**Paper-based stock record** (Paper prototypes)	Most pictures worked well in assisting the CHV to identify the commodity and the symptoms it treats The circles used in counting the commodities helped those who could not write numbers When supply chain terms were explained in Swahili, it increased understanding of the action that CHVs needed to do in reporting If there was a color picture of the actual commodity, this helped CHVs to identify it	Some CHVs had challenges identifying some icons (e.g., family planning method and the avoiding pregnancy icon) CHVs found it difficult to find where to enter their names CHVs were familiar with reporting data horizontally (rows) rather than vertically (columns) Users generally ignored the “units” column and counted based on what they were used to Some users found the shading of circles difficult, making it difficult to total and some recorded by using tally lines through each circle CHVs with low literacy could not identify different columns with supply chain terms Some CHVs entered their age as opposed to the year which the report is being submitted	Changed icons that were confusing to users Moved CHV names and their details to the left of the document Changed Month, to Month of Report and Year of Report to clearly define purpose of cell Included clear visual distinction between commodities such as a gray bar between commodities and the alternate shading of horizontal rows Used horizontal rows instead of columns for each commodity Included colloquial or familiar terms for some commodities (e.g., dewormers for albendazole) Added Swahili translations in brackets under existing supply chain terminology to aid in the translation of often confusing supply chain terminology
**Redesigned cStock smartphone application** (Paper then clickable prototypes)	The audio was the most appreciated feature by the CHVs as it helped identify the commodities The icons allowed for CHVs to identify commodities based on look rather than text The CHVs were also able to identify icons that they had previously seen in the paper recording tool The back and next arrows were understood by some of the CHVs They were able to scroll down the months when selecting the period for reporting	CHVs found some of the icons confusing because they were not representative of reality Some CHVs clicked the icons and not the buttons when selecting which reports to send Most CHVs had difficulty identifying the fields to enter their data Some users understood the arrows to mean “next,” but other users did not	Changed the receiving and dispensing icons based on user feedback Added audio for most icons Changed the icon for audio from a speaker to a person speaking Changed commodity icons to reflect the commodities' actual look Changed icons to match the revised paper tool Included navigation arrows, as well as buttons with “next and back” to allow multiple paths to navigate the app
**CHV supervisor smartphone application** (Paper then clickable prototype)	Supervisors appreciated the prototype The user experience was easy, and they were able to generally follow it with ease The tech was easy to use, even at various levels of technology literacy	Some supervisors were not able to relate how the dashboard related to the paper-based resupply worksheet The validation section of the app was confusing due to unclear wording Most CHVs found the section added for additional CHVs (CHVs that migrate into your area) confusing	Made it clearer that resupply values were the total amounts received from the facility Changed column heading for the checkboxes in the validation page Relabeled additional CHV section in dashboard to articulate that those are CHVs who have migrated to the area
**Hybrid USSD and SMS reporting system** (Paper prototype)	A large number of the CHVs interviewed had a feature phone as their main phone	This prototype was the most difficult for all CHVs, both literate and low literate CHVs were not able to switch from USSD to SMS. They entered data on the dial pad and not the SMS	Needed to be redesigned to only USSD and significant tests were done to ascertain its usability in these communities

Abbreviations: CHV, community health volunteer; SMS, short message service; USSD, unstructured supplementary service data.

Usability testing revealed that CHVs were able to learn how to use a simpler digital solution that used more visual cues and required less steps.

Further research is required to fully understand the benefits of HCD in the design of supply chain and digital health solutions for this population. Its benefits might include fewer post-implementation iterations of tools, better uptake and acceptance of solutions, and improved sustainability. As the tools and systems are rolled out to these communities in the ASAL counties of Kenya over the next year, HCD will continue to be used during the process of implementation. The design team will continue to observe and listen to the users, and will adapt solutions and processes to address their needs—and will then observe the longer-term benefits and potential cost savings of using the HCD process prior to implementation.

### Limitations

HCD has some limitations. Compared to other program design approaches, HCD and systems analysis can take a greater time investment up front (in this case it took 7 months). The approach requires significant time before implementation to conduct the research (2 to 3 months), thoroughly understand the end users (and establish trust with them), and develop and repeatedly test the design and high-fidelity prototypes of the solutions (2 to 3 months). This HCD process was longer than an average process, as it did focus on a particularly complex environment, and 4 different solutions were designed, tested, and iterated. This long lead time can result in some frustration for users and partners who are enthusiastic about finding solutions to their challenges. However, the alternative of quickly developing a product and then testing usability may result in the solution requiring major changes since it was designed *for* the user but not *with* the user.^17^ HCD, which centers on designing digital solutions in complex environments, may potentially result in less time spent overall since uptake is faster and more intuitive and fewer iterations to the tools are required after implementation; however this needs to be evaluated.^18^

HCD when implemented in its purest form can take a long time and is resource-intensive. However, there are different ways to integrate HCD in a project that could shorten the time for the design process. In this instance, it was combined with training project staff in the methodology; alternatively, an experienced external design team could have been hired and the process would be faster but more expensive. It should be noted that HCD requires specialized expertise to lead the process, and individuals working in governments of low- and middle-income countries may not have these skills. At this time, HCD is a specialized methodology that requires external assistance. HCD is most suitable to solve complex problems, such as the one described in this article, where previous health interventions have failed.

In this project, we combined HCD with system analysis and supply chain design methodologies that supported the design team to also consider a wider system strengthening perspective.^19^ We saw this as necessary in our highly complex social and cultural context. However, this approach has the potential disadvantage that it results in a highly context-specific solution that may not be applicable in other contexts. For this project, the redesigned cStock application and USSD is being implemented in Siaya County; this will provide valuable insights into whether the redesigned cStock is well adopted in more traditional community health units or if it is highly context-specific to the ASAL counties. HCD can also be narrow in focus, converging on certain complex problems and specific user challenges and not focusing on the larger system challenges. Another limitation of HCD is that it does not entail rigorous research nor does it provide quantitative data for measuring the impact or outcomes of the program. As with other social and behavioral methodologies, HCD may need to be complemented by traditional evaluation methodologies to generate evidence of program effectiveness.

## CONCLUSION

CHVs in marginalized, nomadic communities live and work in a complex environments. Health services and tools need to be designed to address the unique needs of these communities if health systems are to improve universal access to health care and reduce health inequities. By incorporating the voice of CHVs and their communities, HCD approaches facilitated the design of innovative digital health solutions to support a data-driven supply chain that reflected the needs and expectations articulated by the CHVs and thus is more likely to be used and owned by CHVs. Further evaluation is required at the end of project to assess and reflect on the value of HCD in designing effective digital solutions that have high rates of uptake in complex settings. To ensure that the larger goal of improving access to health commodities and contributing to improved health outcomes of the population is reached, an evaluation should also consider standard performance metrics to assess the supply chain and availability of essential medicines.^20^

## Notes

**Peer Reviewed**
